# Diagnostic value of prehospital arterial blood gas measurements – a randomised controlled trial

**DOI:** 10.1186/s13049-019-0612-8

**Published:** 2019-03-18

**Authors:** Stine T. Zwisler, Yecatarina Zincuk, Caroline B. Bering, Aleksander Zincuk, Mads Nybo, Søren Mikkelsen

**Affiliations:** 1 The Mobile Emergency Care Unit, Department of Anaesthesiology and Intensive Care, Odense University Hospital, J.B. Winsløws Vej 4, DK-5000 Odense, C Denmark; 2 Department of Anaesthesiology and Intensive Care, Odense University Hospital, DK-5000 Odense, C Denmark; 3 Department of Clinical Biochemistry and Pharmacology, Odense University Hospital, DK-5000 Odense, C Denmark; 4 Institute of Regional Health Research, University of Southern Denmark, DK-5000 Odense C, Denmark

**Keywords:** Blood gas analysis, Emergency medical services, Point-of-care analysis

## Abstract

**Background:**

Arterial blood gas analysis is an important diagnostic tool in managing critically ill patients within the hospital. Whether prehospital application of this diagnostic modality contributes to more exact diagnoses and treatments in critically ill prehospital patients is unknown. The aim of this study was to establish whether access to arterial blood gas analysis increased the prehospital diagnostic accuracy of prehospital anaesthesiologists. Furthermore, we investigated whether prehospital blood gas analysis resulted in therapeutic interventions that would not have been carried out if the arterial blood gas analyser had not been available.

**Methods:**

In a prospective randomised study, two groups of prehospital adult patients with acute critical illness were compared. All patients received standard prehospital care. In the intervention group, an arterial blood gas sample was analysed prehospitally. The primary outcome was the impact of blood gas analysis on the accuracy of prehospital diagnoses. Furthermore, we registered any therapeutic interventions that were carried out as a direct result of the blood gas analysis.

**Results:**

A total of 310 patients were included in the study. Eighty-eight of these patients were subsequently excluded, primarily due to difficulties in obtaining post hoc consent or venous sampling or other technical difficulties. A total of 102 patients was analysed in the arterial blood gas group (ABG group), while 120 patients were analysed in the standard care group (noABG group). In 78 of the 102 patients in the ABG group, the prehospital physician reported that ABG analysis increased their perceived diagnostic precision. In 81 cases in the noABG group, the lack of arterial blood gas analysis was perceived to have decreased diagnostic accuracy. The claim that ABG analysis increased diagnostic accuracy could, however, not be substantiated as there was no difference in the number of un-specific diagnoses between the groups.

Blood gas analysis increased the probability of targeting specific prehospital therapeutic interventions and led to 159 interventions, including intubation, ventilation and/or upgrading the level of urgency, in 71 ABG-group patients (*p* < 0.001).

**Conclusion:**

Although prehospital arterial blood gas analysis did not improve the accuracy of the prehospital diagnoses assigned to patients, it significantly increased the quality of treatment provided to patients with acute critical illness.

**Trial registration:**

ClinicalTrials.gov, NCT03006692, retrospectively registered six months after first patient entry.

## Introduction

Impaired consciousness is sometimes considered a non-specific complaint, and this may reduce the quality of treatment and diagnosis in this group of patients as it has been reported that treatment and diagnosis are not as effective in patients with non-specific complaints as they are in awake patients [1]. The diagnostic uncertainty associated with acutely ill patients has been termed the “hallmark of emergency medicine”. Emergency physicians are thus challenged by the vast spectrum and acuity of clinical presentations they are required to diagnose in a data-poor, rapidly evolving, decision-dense environment [2]. This statement probably holds true in the prehospital environment as well as prehospital diagnostic capabilities are usually less comprehensive than those used within the hospital.

In previous decades, point of care analyses of biomarkers are increasingly available in the prehospital setting. For many years, blood glucose measurement has been possible in every ambulance in Denmark, and measurements of troponin or lactate can a be performed prehospitally [3, 4]. The use of ultrasonography has increased diagnostic accuracy [5–7], and mobile prehospital CT scanners are currently being tested for use in patients with suspected cerebral thrombosis to facilitate immediate prehospital thrombolysis [8, 9].

The concept of performing prehospital arterial blood gas (ABG) analysis is not yet widespread. Some older studies have reported that it can be difficult to obtain arterial blood samples in critically ill prehospital patients, and the associated analytical equipment was shown to have a considerable failure rate [10–12]. More recent studies have, however, demonstrated that techniques have improved [13]. Additionally, emerging reports indicate that the ability to target patient treatment has improved [14–16]. Studies utilising prehospital ABG analysis have established that the correlation between end-tidal carbon dioxide and arterial carbon dioxide is poor in critically ill patients needing artificial ventilation and oxygenation [17].

Reduced consciousness can be caused by a large array of underlying conditions ranging from acute respiratory or metabolic disturbances to infection, sepsis, intracranial catastrophe, or injury. The tentative diagnosis made prehospitally without access to ABG analysis is not always accurate [18]. Upon patient arrival at the hospital, ABG analysis is often the first diagnostic adjunct used to supplement a basic evaluation of respiratory and circulatory parameters. We therefore hypothesised that performing ABG analysis prehospitally would aid the prehospital physician in the diagnostic process and increase the probability of targeted therapeutic interventions being applied.

The ABL-90 blood gas analyser (Radiometer®, Denmark) was implemented in the Mobile Emergency Care Unit (MECU) in Odense, Denmark in 2014 [19]. The MECU covers a large mixed urban and rural area and primarily admits patients to the regional university hospital. The MECU has approximately 10 emergency runs per day [20]. In daily in-hospital practice, the use of ABG analysis is usually performed at the discretion of the attending physician. Currently, the MECU in Odense is the only anaesthesiologist-manned ground-based or airborne prehospital unit in Denmark that has access to prehospital ABG analysis.

To investigate the potential for improving the quality of prehospital treatment, the primary aim of this study was to determine whether access to prehospital ABG analysis resulted in a perception of greater diagnostic accuracy by prehospital anaesthesiologists. Furthermore, a comparison between the prehospital “field” and the final (discharge) diagnoses was carried out for each patient. Second, we sought to evaluate whether enabling prehospital anaesthesiologists to perform ABG analysis would impact critical prehospital treatment decisions by registering therapeutic interventions that were carried out as a direct result of the ABG analysis.

## Methods

### Study design

This single-centre, prospective randomised controlled trial was conducted at the MECU in Odense, Denmark. The MECU is an anaesthesiologist-manned rapid-response vehicle in which an emergency medical technician with special training assists one of thirteen specialists in anaesthesiology. The prehospital duration of experience of the anaesthesiologists ranged from 1 ½ years to more than 20 years. The MECU is available around the clock all year. It is not equipped to transport patients, but when dispatched, it acts as an extra resource to supplement the primary ambulance. The MECU covers a population of 260,000 individuals living in an area of 2500 km^2^. The MECU is dispatched in approximately one-quarter of all emergency missions requiring an ambulance [20].

Within the period of June 2016 to April 2018, critically ill adult patients (defined as patients with impaired mental status (Glasgow Coma Score (GCS) < 13)) who were attended by the MECU were randomised prehospitally for either ABG analysis or no ABG analysis. Both groups received standard prehospital anaesthesiologist-directed care. Patients in whom the urgency or extent of the necessary therapeutic interventions precluded a randomisation process with ensuing blood sampling were excluded. Thus, patients in need of immediate therapeutic intervention who required ongoing therapeutic interventions en route to the hospital were not included if their inclusion interfered with immediate care. Furthermore, patients with a GCS ≥13 and those who were < 18 years old, pregnant and/or breastfeeding, or detained by the police or under compulsory treatment were also excluded. A further exclusion criterion was permanent incapacitation prior to the specific critical illness. Finally, patients in whom informed consent could not be obtained post hoc as per the approved protocol were excluded.

A proxy parameter for good quality in treatment was the accuracy of the diagnoses. The sample size was thus calculated on the basis of the expected occurrence of patients assigned the least specific diagnosis in the International Statistical Classification of Diseases and Related Health Problems 10th Revision (ICD-10) system (i.e., diagnosis code Z03.9; observation for suspected disease or condition, unspecified) [20]. The expected occurrence was 4%. The aim was to reduce the number of patients with this particular diagnosis code to 3.6%. The probabilities of type I and type II errors were set at 0.05 and 0.20, respectively. Based on these numbers, 98 patients were expected in each group. To allow drop-outs, we planned to include 110 patients in each group.Randomisation took place at the prehospital scene during the initial assessment but after all emergency procedures, such as immediate airway management or initiating resuscitation, were completed. When the inclusion criteria were met, randomisation was performed by opening an envelope containing instructions for allocation. Equal numbers of opaque envelopes were packed individually for each group with a random allocation sequence of 5:5. The 10 envelopes were mixed into random sequences and packed by a secretary with no relation to this study. In individuals allocated into the intervention group, an arterial blood sample of 0.5–1.0 ml was drawn from the femoral or radial artery and analysed immediately on-site using an ABL-90 blood gas analyser (®Radiometer, Denmark) [19]. The result was available within minutes and enabled the anaesthesiologist to make adjustments to the patient’s prehospital treatment.After the completion of the mission, the anaesthesiologist prepared a discharge summary, and the patient was assigned a tentative diagnosis according to ICD-10 [21]. The anaesthesiologist also evaluated whether the availability of ABG analysis was significant in relation to the decisions made regarding and the treatment of the patient and/or the accuracy of the diagnosis.

### Questionnaire

The prehospital anaesthesiologist filled in a case report form (CRF) for each randomised patient after finishing prehospital care and management. The following questions were answered:

- Allocation group according to ABG analysis or no ABG analysis;
- Diagnosis based on the ICD-10 classification;
- Documentation of data obtained from the ABG, including pH, pCO_2_, pO_2_, base excess, oxygenation (SpO_2_), haemoglobin (Hgb), electrolytes (including potassium (K^+^), sodium (Na^+^), bicarbonate (HCO_3_^−^) and calcium (Ca^2+^)), glucose and lactate;
- A description of any interventions performed following the completion of emergency procedures that were initiated as a result of the ABG analysis; i.e., correction of acidosis, administration of fluids, tracheal intubation, and other interventions;
- To what degree the anaesthesiologist found that the ABG analysis or the lack of ABG analysis influenced the treatment of the patient (crucial importance, major importance, moderate importance, minor importance, no importance); and
- An evaluation of whether the anaesthesiologist perceived the ABG analysis or lack of it to have influenced their general diagnostic accuracy (more precise, minor change in precision, no change in precision).

### Informed consent

Informed consent was obtained retrospectively according to Danish Law 593, §4, no. 3 concerning research in acute, critically ill, incapacitated patients unable to give informed consent before randomisation. Patients were contacted after the initial treatment and stabilisation of an acute illness when they no longer suffered from an impairment of consciousness. In cases in which a patient was permanently mentally incapacitated or dead after the incident, according to the same Danish law, consent to use the collected data was obtained from the closest relative and the patient’s general practitioner. Informed consent was preceded by oral and written information.

This study was approved by the Danish Data Protective Agency, journal number 2012-58-0018, and the Regional Committee on Health Research Ethics, id S-20160032 according to Danish law. The study is registered at ClinicalTrials.gov as NCT03006692.

### Statistics

All data and the CRF entries were manually entered in an Excel sheet (MS Excel, Microsoft Corp., Redmond, WA, USA) and analysed in STATA® 15.1 (StataCorp, LLC, Texas, USA). Background data, including age and GCS, are presented as medians with quartiles.

Data regarding the precision of diagnoses were compared using the Chi-Square test. Data from the Likert-type questionnaires were ranked from negative values through zero to positive values and compared using the Kruskal-Wallis test or Mann-Whitney test, as appropriate. For all analyses, a *p*-value of < 0.05 was assumed to be statistically significant.

## Results

**Fig. 1:**
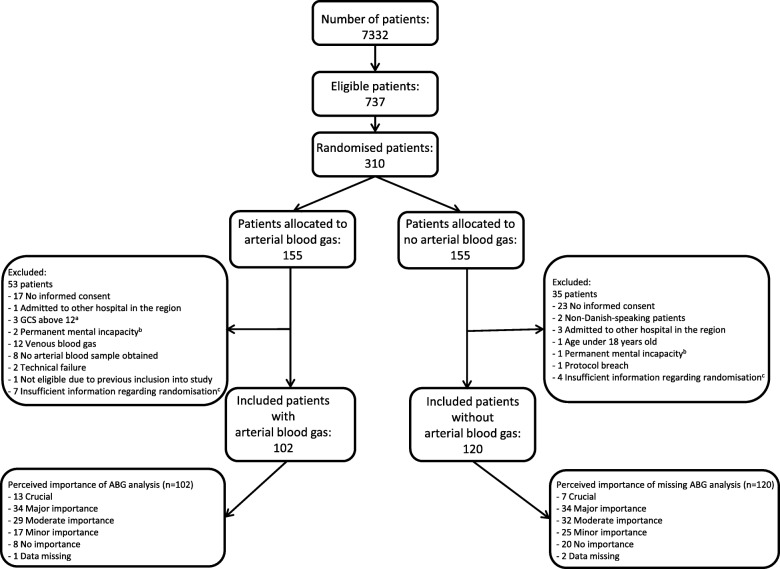
Flowchart of patients. **a**: Glasgow Coma Score. **b**: Permanent mental incapacitation leading to a habitually low GCS. **c**: Insufficient randomisation information: Randomisation envelopes not included in the Case Report Forms

During the 22-month study period, the MECU was involved in 7332 missions that treated a total of 737 patients with a GCS of 13 or below who needed to be admitted to hospital with a physician escort. Of these patients, 310 were included in the study. In the ABG group and the noABG group, 53 and 35 patients, respectively, were excluded before data analysis. Exclusion was primarily due to the research group being unable to obtain informed consent following the incident. In the ABG group, 20 patients were excluded either because the anaesthesiologist drew the venous samples on-site (12 patients) or because there were technical difficulties in obtaining the sample (8 patients). In total, 102 patients were randomised for ABG analysis, and 120 patients were randomised for no ABG analysis in this study (Fig. 1).

Patients in the two groups were comparable with regard for age, sex, and GCS (Table 1).

**Table 1:** Demographics of the prehospital patients

	Arterial blood gas analysis *N* = 102	No arterial blood gas analysis *N* = 120	*p*-value
Sex (Female/Male) (N)	46/56	56/64	0.92
Age in years (Median/Quartiles)	71 (59–79)	70 (60–83)	0.72
Glasgow Coma Score (Median/Quartiles)	6 (3–9)	6 (3–10)	0.49

### Diagnostic accuracy

The prehospital anaesthesiologists reported that in 78 of the 102 patients analysed in the ABG group, the opportunity to perform this analysis increased their prehospital diagnostic precision. Among the 120 patients randomised for no ABG analysis, in 35 cases, the lack of the potential to perform ABG analysis was not perceived to have altered the accuracy of the prehospital diagnosis, while in 81 cases, the lack of access to prehospital ABG analysis was perceived to have decreased the diagnostic accuracy of the prehospital anaesthesiologist (information was missing from a total of three cases) (Fig. 1). In 76% of the patients with ABG, the attending physicians thus perceived that ABG analysis increased their diagnostic accuracy, whereas in 68% of the patients in the noABG group, the physician felt that the lack of ABG analysis decreased the diagnostic accuracy. This reported difference was significant (*p* = 0.001). We were, however, unable to objectively support the claim that ABG analysis led to more precise diagnoses as there was no difference between the two groups in the assessed primary effect parameter, the numbers of patients in the two groups that were prehospitally assigned the ICD-10 diagnosis code Z03.9 (observation for suspected disease or condition, unspecified). A total of 11 patients were in this diagnosis group (three in the ABG group and eight in the noABG group), and the difference was not significant (*p* = 0.20). Our analysis of the total number of patients assigned a prehospital diagnosis within ICD-10 Chapter XXI (factors influencing health status and contact with health services; eight patients and 13 patients in the ABG and noABG groups, respectively) showed that there was no difference between the two groups (*p* = 0.58). When both the ICD-10 classification system chapters of diagnoses of lesser specificity were combined (Chapters XVIII (Symptoms, signs and abnormal clinical and laboratory findings, not elsewhere classified) and Chapter XXI), our analysis confirmed that there was no difference in diagnostic accuracy between the ABG and noABG groups (28 versus 30 patients, respectively; *p* = 0.46). Importantly, the tentative prehospital diagnosis was identical to the diagnosis assigned to the patient following a full in-hospital work-up in only 55% of the patients (*p* = 0.50; see Table 2).

**Table 2:** Distribution of prehospital and in-hospital diagnoses

ICD-10 chapter	Diagnosis group		Prehospital diagnosis		In-hospital diagnosis
			With ABG (*n* = 102)	Without ABG (*n* = 120)	(*n* = 222)
Chapter I	Certain infectious and parasitic diseases	(A00-B99)	6	4	5
Chapter II	Neoplasms	(C00-D48)	0		6
Chapter III	Diseases of the blood and blood-forming organs and certain disorders involving the immune mechanism	(D50-D89)	0		0
Chapter IV	Endocrine, nutritional and metabolic diseases	(E00-E90)	3	3	8
Chapter V	Mental and behavioural disorders	(F00-F99)	0		2
Chapter VI	Diseases of the nervous system	(G00-G99)	8	7	26
Chapter VII	Diseases of the eye and adnexa	(H00-H59)	0		0
Chapter VIII	Diseases of the ear and mastoid process	(H60-H95)	0		0
Chapter IX	Diseases of the circulatory system	(I00-I99)	33	38	70
Chapter X	Diseases of the respiratory system	(J00-J99)	15	22	39
Chapter XI	Diseases of the digestive system	(K00-K93)	2	1	5
Chapter XII	Diseases of the skin and subcutaneous tissue	(L00-L99)	0		0
Chapter XIII	Diseases of the musculoskeletal system and connective tissue	(M00-M99)	0		0
Chapter XIV	Diseases of the genitourinary system	(N00-N99)	0	3	4
Chapter XV	Pregnancy, childbirth and the puerperium	(O00-O99)	0		0
Chapter XVI	Certain conditions originating in the perinatal period	(P00-P96)	0		0
Chapter XVII	Congenital malformations, deformations and chromosomal abnormalities	(Q00-Q99)	0		0
Chapter XVIII	Symptoms, signs and abnormal clinical and laboratory findings, not elsewhere classified	(R00-R99)	20	17	26
Chapter XIX	Injury, poisoning and certain other consequences of external causes	(S00-T98)	9	10	22
Chapter XX	External causes of morbidity and mortality	(V01-Y98)	0		0
Chapter XXI	Factors influencing health status and contact with health services	(Z00-Z99)	8	13	5
Chapter XXII	Codes for special purposes	(U00-U85)	0		0
Missing					4

### Therapeutic interventions

In 86 of the 102 patients randomised to the ABG group, ABG as a supplemental diagnostic modality was perceived as crucial, of major importance, or of moderate importance for selecting treatment options. In the 120 patients randomised to the noABG group, the anaesthesiologists reported that the presence of ABG analysis would have been crucially important, of major importance, or of moderate importance in 73 cases (*p* < 0.0001). See Table 3.

**Table 3:** Self-reported evaluation of the perceived benefit of arterial blood gas analysis in relation to treatment options

	Perceived benefit of ABG analysis (*n* = 220)	% (95% CI)
Crucial	20	9.0% (5.6–13.6%)
Major importance	68	30.6% (24.6–37.1%)
Moderate importance	61	27.5% (21.7–33.9%)
Minor importance	42	18.9% (14.0–24.7%)
No importance	28	12.6% (8.5–17.7%)
Data missing	3	1.4% (0.3–3.9%)

In 71 of the 86 patients in whom the ABG analysis was considered important for selecting treatment options, this analysis led to a total of 159 interventions, such as ventilation, intubation, or correction of electrolytes. In the remaining 15 patients in whom ABG was reported to have aided the anaesthesiologist in selecting treatment options, information regarding the exact intervention(s) brought about by the use of ABG was not listed.

The interventions carried out as a result of the ABG analyses are listed in Table 4.

**Table 4:** Therapeutic interventions resulting from the arterial blood gas analysis

Number of interventions (*n* = 159)	Correction according to ABG
13	PaO_2_
28	PaCO_2_
1	Antidote
1	Glucose
26	Correction of Acidosis (metabolic or respiratory)
6	Correction of electrolytes
22	Ventilation
10	Intubation
3	Refraining from intubation
9	Substitution of fluids
5	Upgrade of level of emergency
7	Downgrade of level of emergency
7	Direct admission to treatment facility other than emergency department
3	Sepsis treatment including blood sampling for bacteria culturing
7	Unspecific medications
11	Miscellaneous interventions^a^

^a^Miscellaneous interventions: Carbon monoxide measurement for the purpose of assessing the need for hospital admission, treatment withheld because of moribund patient or medical case history, or treatment withheld because of confirmation of the patient being in their habitual condition

## Discussion

The prehospital anaesthesiologists reported that the opportunity to supplement basic prehospital diagnostic procedures with ABG analysis improved the precision of the diagnoses assigned to the patients. This claim could not be confirmed because there was no difference in the number of patients who were assigned a diagnosis of lesser specificity between the groups of patients, and there was no difference in the precision of the diagnoses assigned to the patients in the two groups. However, the prehospital anaesthesiologists reported that the prehospital ABG analysis provided them with better treatment options. In 13 cases, the physicians reported that for various reasons, they found it crucial to have access to the ABG analysis. In a further seven cases in which the patient had been randomised to the noABG group, the anaesthesiologists reported that access to the ABG analysis would have been crucial. Indeed, in one case, the physician refused to randomise a patient because he insisted that he could not ethically defend not having an ABG analysis performed. The patient was a trauma patient with facial fractures and an impaired airway who had been lying on the floor for hours and hence was at risk of developing hyperkalaemia. The attending physician found it crucial to know the level of potassium before choosing the muscle relaxant for intubation. Hence, this particular patient represented a naturally occurring protocol breach and was not included in the study.

In general, the ABG analysis results seemed to aid the prehospital anaesthesiologists in selecting therapeutic interventions. ABG analyses were applied in clinical use in cases involving patients with cardiac arrest, in whom a finding of high levels of potassium could enable the prehospital anaesthesiologist to apply potassium-reducing treatments or aid them in terminating treatment. In other cases, the finding of severe metabolic acidosis in patients suffering from cardiac insufficiency enabled the prehospital physician to direct patients for immediate interventional cardiology procedures rather than admission to the wards.

In 69.6% of the cases in which the patient was subjected to ABG analysis, the analysis led to a specific therapeutic intervention. This is comparable to the findings presented in a previous study by Wildner et al., in which 77.8% of the members of a mixed prehospital patient group were offered therapeutic changes according to the ABG result [13].

In the seven patients allocated to the noABG group in whom the prehospital anaesthesiologist reported that the ABG analysis would have been crucial for treatment, the physicians reported that they lacked the diagnostic support necessary to administer specific treatment, i.e., antibiotics in septic patients, or to direct the patient to a specific department at the hospital.

The use of ABG is not without challenges. Twenty years ago, the use of different blood gas analysers was evaluated. It was reported that even the most reliable blood gas analyser at that time resulted in technic failure in approximately 20% of all applications [11]. Even when used by trained anaesthesiologists, procedural difficulties were encountered when attempting to draw an arterial blood sample in 10 out of 36 cardiac arrest patients undergoing resuscitation [9]. In a more heterogeneous population of critically ill patients, 34% of the arterial blood gas analyses could not be performed due to technical failure [12]. In another study that investigated early ABG analysis in traumatised patients, the arterial blood sample was not analysed until arrival at the hospital. Despite the fact that it investigated early ABG analysis, that study provided no additional prehospital diagnostic tools [22]. In our study, technical difficulties were experienced in obtaining ABG in a total of 22 patients (7%), including twelve patients in whom venous blood samples were erroneously drawn, eight patients in whom it was not possible to obtain a blood sample, and two patients in whom the ABL analyser failed. One could argue that this failure rate was, when considering the amount of useful clinical information obtained in our study, for practical purposes somewhere between 3 and 7%. This assumption is based on the fact that venous blood, in addition to providing information regarding oxygenation, also provides some information regarding acidity, lactate, and other biomarkers. The eight patients in whom it was not possible to obtain any blood sample were mostly patients in cardiac arrest in whom arrest interchanged with spontaneous circulation multiple times during prehospital care and transport. In our study, the failure rate of obtaining an analysis of an ABG sample during ongoing cardiac arrest was similar to the rate reported in a prospective study on ABG sampling performed in patients with cardiac arrest [15]. That particular study demonstrated that not only are patients in cardiac arrest characterised by metabolic acidosis, but they also experience clear hypoventilation based on their blood gas profiles [15].

Previous studies have indicated that lactate measurement may be used as a tool for screening patients at higher risk of mortality or those with a higher risk of admission to the intensive care unit, but in these studies, no attempt was made to link the lactate measurements to diagnostic accuracy or the initiation of prehospital treatment other than fluid administration [23, 24]. In our study, obtaining a complete ABG analysis provided more information about the patient’s general condition than can be obtained via lactate analysis alone. Thus, rather than using point-of-care analyses as a screening tool, we investigated whether the available test results increased the diagnostic and therapeutic capabilities of the prehospital physicians.

According to our predefined research question, our primary effect parameter was diagnostic accuracy. Although the prehospital anaesthesiologists reported that having access to ABG analysis increased diagnostic accuracy, this finding could not be confirmed as only 55% of the diagnoses assigned to the patients remained unaltered following in-hospital examinations. However, the primary reason that patients were ultimately assigned to a diagnosis group other than the prehospital diagnosis group was an in-hospital transfer from the ICD-10 Chapters XVIII and XXI to more specific diagnosis chapters. This apparent reluctance towards assigning a firm diagnosis may be supported by a study by Heuer et al., who stated that prehospitally, it may be better to assign a patient no diagnosis than to assign the patient an erroneous diagnosis [18]. However, when investigating prehospital ABG analysis as a tool for determining therapeutic needs in patients, ABG analysis was found to be useful. In three-quarters of the patients, the attending prehospital anaesthesiologists found that ABG analysis provided them with a moderate, major, or crucially better foundation on which to target specific therapeutic interventions in conscious-impaired prehospital patients.

### Limitations

In-hospital, the use of ABG analysis has been implemented as a standard evaluation for critically ill patients. Thus, with respect for the perceived overall benefit of ABG analysis, the finding that access to ABG analysis was considered beneficial should come as no major surprise. Several other factors limit the validity of this study. One limitation is that the study was not blinded. The anaesthesiologist in charge of the treatment and the anaesthesiologist that evaluated the clinical importance of the ABG analysis were the same person.

Another limitation is that some patients were excluded due to the urgency and extent of the necessary critical interventions. These patients may have been patients in whom an ABG analysis could in essence could have been considered important. However, in cases in which the tasks of the prehospital physician were too urgent and too many, so that they had no time to conduct a sampling for an ABG analysis, one may assume that the necessary critical interventions were carried out based on less subtle parameters than those provided by the ABG analysis alone.

Other limitations include the apparent lack of reporting of the influence of the ABG analysis on treatment. Although the physicians reported that the treatment of 86 patients benefitted from ABG analysis, in 15 patients, the exact benefit in terms of which specific therapeutic interventions the ABG analysis resulted in was not reported. This influences the validity of the results.

A further limitation of this study is that post hoc consent could not be obtained in all patients who remained incapacitated following the prehospital event. However, we suggest that this is not likely to have resulted in systematic bias as the patients were randomised without any consideration of the precipitating cause of the temporarily impaired mental status.A minor limitation of this study is that it was a single-centre study. Its external validity might have been increased if we had been able to include other similar ground-based, anaesthesiologist-manned rapid response vehicles. However, this was not possible because the MECU of Odense is the only Danish prehospital unit presently able to perform ABG analyses. Finally, a limitation is that the CRF was filled out by the anaesthesiologists following the event. Thus, an element of recall bias may have been introduced that we were not able to control.

### Strengths

The main strength of this study is that it was prospectively conducted with a predefined hypothesis. A further strength of the study is that we not only investigated the prehospital anaesthesiologists’ perception of the benefit provided by the availability of ABG analysis but also investigated to what extent the ABG analysis led to a treatment modality that was not initiated before the result of the ABG analysis was known.

## Conclusion

In conclusion, we could not confirm that the ability of the prehospital anaesthesiologists to assign the patient a more precise prehospital diagnosis was better when the prehospital anaesthesiologist had access to ABG analysis than when they did not. The prehospital anaesthesiologists reported that having access to ABG analysis provided them with a better foundation for making decisions regarding treatment in conscious-impaired prehospital patients. This outcome was supported by the finding that in three-quarters of the patients in the ABG group, the test result led to specific therapeutic interventions.

## Abbreviations

ABG: Arterial Blood Gas
GCS: Glasgow Coma Score
ICD-10: International Statistical Classification of Diseases and Related Health Problems, 10th Revision
MECU: Mobile Emergency Care Unit
